# Artificial intelligence for affective-domain development in healthcare professions education: a systematic review

**DOI:** 10.3389/fmed.2026.1894820

**Published:** 2026-07-30

**Authors:** Noor Akmal Shareela Ismail, Azizah Zafira

**Affiliations:** Faculty of Medicine, Universiti Kebangsaan Malaysia, Kuala Lumpur, Malaysia

**Keywords:** affective-domain learning, artificial intelligence, communication skills, health professions education, virtual patient

## Abstract

**Background:**

Artificial intelligence (AI) is increasingly integrated into health professions education through generative AI, chatbots, virtual patients, automated feedback, and AI-enhanced simulation. While most discussions focus on cognitive and technical learning, the role of AI in affective-domain development remains less clearly understood. Affective-domain learning is essential in healthcare education because it shapes communication, empathy, professionalism, reflection, self-awareness, and patient-centered practice.

**Objective:**

This systematic review examined how AI-based educational tools and interventions have been used to support affective-domain development among healthcare professional students and trainees.

**Methods:**

This review followed PRISMA 2020 guidance. Searches were conducted in PubMed, Scopus, Web of Science, SpringerLink, ScienceDirect, Google Scholar, Wiley Online Library, and IEEE Xplore for English-language empirical studies published between January 2010 and May 2026. Eligible studies involved healthcare professional learners, used AI-based educational tools for teaching, simulation, feedback, communication training, reflection, or assessment, and reported at least one affective-domain-related outcome. Methodological quality was appraised using Joanna Briggs Institute critical appraisal tools as the primary appraisal framework, with CASP used only as a supplementary interpretive aid for studies with qualitative or mixed-methods components. Findings were synthesized narratively because of heterogeneity in study designs, AI interventions, and affective-domain outcomes.

**Results:**

Seventeen studies were included involving undergraduate and post-graduate healthcare learners. AI tools were primarily used for communication training, virtual patient interaction, reflective feedback, breaking-bad-news simulation, medical interview rehearsal, and OSCE-style assessment. Most studies reported improvements in learner confidence, self-efficacy, reflective engagement, perceived communication skills, or simulation-based assessment performance. AI was valued for providing repeated low-stakes practice, immediate structured feedback, accessibility, and scalability. However, evidence supporting deeper affective outcomes such as empathy, emotional responsiveness, relational authenticity, and non-verbal communication remained limited. Several studies also highlighted concerns regarding emotional realism, over-reliance on AI, and reduced interpersonal authenticity.

**Conclusion:**

AI may support selected aspects of affective-domain learning, particularly communication rehearsal, reflective learning, and formative feedback. However, AI should complement rather than replace human-facilitated teaching, as educators remain essential for fostering empathy, ethical judgement, professional identity formation, and relational care.

**Systematic review registration:**

https://www.crd.york.ac.uk/PROSPERO/view/CRD420261401425, Identifier: CRD420261401425.

## Introduction

Artificial intelligence (AI) is increasingly embedded in healthcare and health professions education through learning support, assessment, simulation, clinical reasoning, automated feedback, academic writing, and virtual patient interaction (1, 2). Recent advances in generative AI and large language models (LLMs) have further accelerated this integration by enabling conversational interfaces, adaptive feedback, and more realistic simulated clinical dialogue beyond traditional classroom and clinical environments (3–5). These technologies may support scalable, personalized, and flexible learning, particularly where faculty time, simulated patient access, clinical placements, and formative feedback opportunities are limited (6, 7).

Much of the literature on AI in medical and health professions education has focused on cognitive and technical learning, including diagnostic reasoning, knowledge acquisition, procedural performance, assessment, and examination outcomes (3, 4). However, healthcare education also requires affective-domain competencies, including empathy, communication, professionalism, ethical judgement, self-awareness, accountability, reflective capacity, leadership, and patient-centered values (8, 9). These competencies are essential to safe, compassionate, and socially responsive healthcare because they shape how professionals relate to patients, families, colleagues, and wider healthcare systems (8, 10).

The affective domain refers to attitudes, values, emotions, motivations, and professional dispositions that influence behavior and decision-making in practice (9). Bloom's taxonomy and Krathwohl's affective-domain framework conceptualize affective learning as a progression from receiving and responding to phenomena, toward valuing, organization, and internalization of professional values and behaviors (11). In health professions education, this framework supports the development of empathy, professionalism, ethical sensitivity, reflective capacity, communication, and patient-centered care (8–11). Unlike cognitive learning, affective-domain learning involves professional identity formation, interpersonal values, emotional awareness, and socially responsive behavior, and therefore requires experiential learning, guided reflection, feedback, mentorship, and authentic human interaction (8, 9, 12).

This review was conceptually guided by Krathwohl's affective-domain framework (11). AI-supported affective-domain training was interpreted in relation to this developmental progression, with attention to communication behaviors, reflective engagement, empathy, professionalism, confidence, self-efficacy, patient-centered interaction, and learner preparedness (8–11). This framing reflects the “ways of being” expected of healthcare professionals, including communication, collaboration, reflective practice, ethical reasoning, autonomy, accountability, social responsibility, cultural sensitivity, and systems awareness (9, 10, 12–14). Affective-domain development is therefore not an optional supplement to healthcare education, but a core dimension of professional formation (8, 9).

Despite its importance, affective-domain development remains difficult to teach and assess. Competencies such as communication, empathy, professionalism, and reflective capacity require repeated exposure, emotional engagement, guided reflection, feedback, and opportunities to practice in authentic or near-authentic contexts (8, 9). Traditional approaches, including bedside teaching, role-play, simulated patients, reflective writing, mentorship, and facilitator feedback, are valuable but resource-intensive, labor-dependent, and difficult to deliver consistently across large learner cohorts (7, 8). Variability in clinical exposure, faculty expertise, and feedback quality further complicates affective-domain training (7–9).

These challenges have increased interest in whether AI-based educational tools can support affective-domain learning by expanding opportunities for practice, simulation, reflection, and feedback (4, 7, 13). Conversational agents, virtual patients, and AI-enhanced simulations may allow learners to rehearse history taking, counseling, breaking bad news, and patient-centered communication in psychologically safe, low-stakes environments (7, 15). AI-generated feedback may also provide immediate and structured guidance on communication behaviors, reflective writing, consultation performance, and professional interactions (15). Compared with conventional rule-based virtual patients, generative AI systems may offer more adaptive encounters that respond to learner questioning styles, emotional prompts, and variations in patient health literacy (3, 4, 7).

However, AI use in affective-domain education raises educational and ethical concerns (4, 14). Affective learning depends on emotional nuance, relational authenticity, empathy, non-verbal communication, contextual judgement, and cultural sensitivity, which AI systems may not reliably reproduce (8, 10, 14). Although AI-generated feedback may be detailed and consistent, it may lack emotional understanding, contextual interpretation, and human relational depth (10, 15). Similarly, virtual patients may provide accessible rehearsal opportunities but may not adequately capture trust, vulnerability, uncertainty, distress, or the embodied emotional presence of real clinical interactions (7, 8). Concerns also remain regarding depersonalization, over-reliance on AI-mediated interaction, and reduced authentic human engagement in professional formation (9, 14).

Although interest in AI-supported affective learning is growing, the literature remains fragmented across communication training, reflective learning, virtual simulation, professionalism education, and assessment. Existing reviews on AI in health professions education have mainly addressed opportunities, implementation, technical applications, or cognitive outcomes, while evidence specific to affective-domain development has not been comprehensively synthesized (3, 4, 7). Affective outcomes are also inconsistently conceptualized and measured, making it difficult to determine the educational role, benefits, methodological quality, and limitations of AI within this domain (8, 9).

To address these gaps, this systematic review aimed to: (1) examine how AI-based educational tools have been used to support affective-domain development among healthcare professional students and trainees; (2) identify the affective-domain outcomes most commonly targeted, including communication, empathy, reflection, professionalism, confidence, self-efficacy, patient-centered interaction, and professional preparedness; and (3) synthesize the reported benefits, limitations, and methodological quality of AI-supported affective-domain training to inform curriculum design, responsible educational implementation, and future research.

## Methods

### Protocol and registration

This systematic review was registered in PROSPERO under the registration number CRD420261401425 and reported in accordance with the PRISMA 2020 statement. The completed PRISMA 2020 checklist is provided in Supplementary File 1. The review was conceptually informed by Bloom's taxonomy and Krathwohl's affective-domain framework. No major deviations from the registered protocol occurred. However, because of substantial heterogeneity in study designs, learner populations, AI interventions, and affective-domain outcomes, meta-analysis was not performed, and findings were synthesized narratively.

### Eligibility criteria

Studies were included if they: (1) involved healthcare professional learners, including undergraduate students, post-graduate trainees, residents, interns, or other trainees in medicine, nursing, pharmacy, dentistry, allied health, or related disciplines; (2) examined an AI-based educational tool or intervention used for teaching, learning, feedback, simulation, reflection, communication training, assessment, or coaching; (3) reported at least one affective-domain-related outcome, such as communication skills, empathy, reflection, professionalism, patient-centered interaction, ethical reasoning, emotional engagement, self-efficacy, learner confidence, or preparedness for patient interaction; (4) used an empirical quantitative, qualitative, mixed-methods, validation, or evaluation study design; (5) was published in English between January 2010 and May 2026; and (6) was available as a full-text peer-reviewed journal article or full conference paper with sufficient methodological and outcome data.

Studies were excluded if they involved general e-learning without AI; conventional virtual reality, augmented reality, or simulation without AI integration; AI tools used only for cognitive, technical, diagnostic, or examination-related outcomes without an affective-domain component; AI literacy, attitudes, acceptance, or readiness without linkage to affective-domain educational outcomes; non-healthcare learners, practicing professionals without a learner context, non-educational settings, or clinical service delivery only; or reviews, meta-analyses, editorials, commentaries, protocols, letters, opinion papers, abstracts only, non-peer-reviewed publications, non-English studies, or studies without accessible full text.

Where eligibility was unclear, studies were assessed according to whether the AI intervention targeted interpersonal, reflective, emotional, professional, or patient-centered learning rather than purely cognitive or technical performance. In this review, the term “learners” was used as an umbrella term to include undergraduate students, post-graduate trainees, residents, interns, and other healthcare professional trainees. The term “students” was used only when referring specifically to undergraduate or pre-licensure student populations. Studies that included non-healthcare stakeholders were retained only when the primary educational context and intervention target were healthcare professional learners or trainees. For example, Armstrong et al. (26) was included because the study focused on post-graduate medical education and breaking-bad-news communication, while lay representatives contributed as evaluators or stakeholders rather than as the primary learner population.

### Information sources

Searches were conducted in PubMed, Scopus, Web of Science, SpringerLink, ScienceDirect, Google Scholar, Wiley Online Library, and IEEE Xplore. The search covered studies published from January 2010 to May 2026, with the final search conducted on 7 May 2026. Only published studies were sought. Unpublished studies, gray literature, trial registries, conference abstracts without full papers, and additional online resources were not systematically searched. Study authors and field experts were not contacted for unpublished or missing data.

### Search strategies

The search strategy was developed using three core concepts: (1) artificial intelligence, (2) health professions education, and (3) affective-domain outcomes. For each concept, pre-defined controlled vocabulary terms, database-specific indexing terms, and free-text keywords were identified before the search was conducted. Terms within the same concept were combined using the Boolean operator OR, and the three concept groups were combined using AND. The search syntax was adapted for each database according to available controlled vocabulary, field codes, phrase searching, truncation, and database-specific Boolean functions.

In PubMed, Medical Subject Headings (MeSH) were combined with title/abstract terms. Relevant MeSH terms included “Artificial Intelligence”, “Machine Learning”, “Natural Language Processing”, “Education, Medical”, “Education, Nursing”, “Education, Pharmacy”, “Communication”, “Empathy”, “Professionalism”, “Patient-Centered Care”, and “Self Efficacy”. Free-text terms included artificial intelligence, generative AI, ChatGPT, chatbot, large language model, LLM, virtual patient, simulated patient, automated feedback, health professions education, medical education, nursing education, pharmacy education, communication skills, empathy, professionalism, reflection, confidence, self-efficacy, patient-centered care, patient-centered care, affective learning, and affective domain.

For example, the PubMed search combined AI-related terms, education-related terms, and affective-domain-related terms as follows: (“Artificial Intelligence”[MeSH] OR “Machine Learning”[MeSH] OR “Natural Language Processing”[MeSH] OR “artificial intelligence”[Title/Abstract] OR “generative AI”[Title/Abstract] OR ChatGPT[Title/Abstract] OR chatbot^*^[Title/Abstract] OR “large language model^*^”[Title/Abstract] OR LLM[Title/Abstract] OR “virtual patient^*^”[Title/Abstract] OR “simulated patient^*^”[Title/Abstract] OR “automated feedback”[Title/Abstract]) AND (“Education, Medical”[MeSH] OR “Education, Nursing”[MeSH] OR “Education, Pharmacy”[MeSH] OR “health professions education”[Title/Abstract] OR “medical education”[Title/Abstract] OR “nursing education”[Title/Abstract] OR “pharmacy education”[Title/Abstract]) AND (“Communication”[MeSH] OR “Empathy”[MeSH] OR “Professionalism”[Title/Abstract] OR “Patient-Centered Care”[MeSH] OR “Self Efficacy”[MeSH] OR “communication skills”[Title/Abstract] OR empath^*^[Title/Abstract] OR reflection[Title/Abstract] OR professionalism[Title/Abstract] OR confidence[Title/Abstract] OR “self-efficacy”[Title/Abstract] OR “patient-centered care”[Title/Abstract] OR “patient-centered care”[Title/Abstract] OR “affective learning”[Title/Abstract] OR “affective domain”[Title/Abstract]).

The search syntax was adapted for Scopus, Web of Science, SpringerLink, ScienceDirect, Google Scholar, Wiley Online Library, and IEEE Xplore using the relevant field codes, phrase searching, truncation, and database-specific Boolean syntax. Where a database did not use MeSH, equivalent indexed terms or database-specific subject terms were used where available and supplemented with free-text terms. The complete database-specific search equations, including Boolean operators, field codes, search dates, and limits, are provided in Supplementary Table 1.

### Study selection

**Figure 1:**
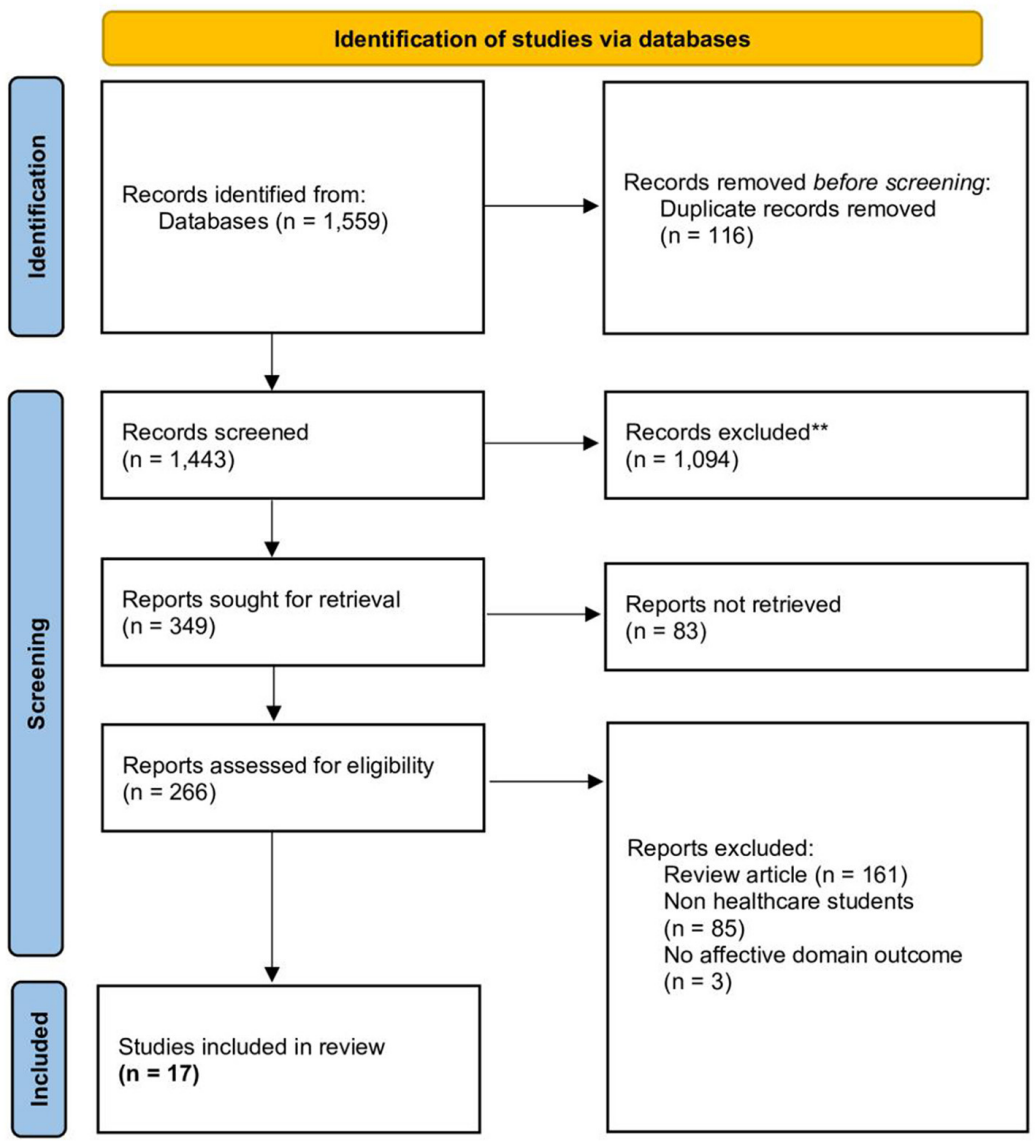
PRISMA 2020 flow diagram of study identification, screening, eligibility assessment, and inclusion. **Records were excluded during title and abstract screening because they did not meet the pre-defined eligibility criteria. Figure was adapted from the PRISMA 2020 flow-diagram template (42), Creative Commons Attribution 4.0 International (CC BY 4.0).

All retrieved records were exported into Microsoft Excel 2020 for search management, and duplicates were removed before screening. Title and abstract screening was conducted independently by two reviewers (AZ and NASI) using pre-defined eligibility criteria. Potentially eligible reports were retrieved for full-text assessment and screened independently by both reviewers. Disagreements or uncertain eligibility decisions were resolved through discussion and consensus. No automation tools were used for eligibility screening. Reasons for exclusion at the full-text stage were documented and summarized in the PRISMA 2020 flow diagram. The study selection process is shown in Figure 1.

### Data collection process

Data were extracted using a standardized form developed according to the review question, eligibility criteria, and planned synthesis domains. Both reviewers independently completed and cross-checked the extraction, with discrepancies clarified during verification. No imputation was performed. Where reporting was incomplete or unclear, extraction was limited to information available in the published manuscript, and study authors were not contacted for additional data.

### Data items and definitions

Extracted data included study characteristics, such as author, year, country, study design, learner population, healthcare discipline, educational setting, sample size, comparator or control where applicable, and study context. Intervention- and outcome-related data included AI tool or intervention type, educational purpose, duration or exposure where reported, affective-domain focus, outcome measures, main findings, learner or faculty perceptions, limitations, and recommendations for future research.

For this review, AI-based educational tools were defined as tools or systems incorporating artificial intelligence to support teaching, learning, feedback, simulation, reflection, communication training, coaching, or assessment. These included generative AI systems, LLM-based chatbots, conversational agents, AI-simulated patients, virtual patients, AI-generated feedback systems, adaptive learning tools, AI-assisted assessment systems, and AI-enhanced simulation. Affective-domain outcomes were defined as learner outcomes related to attitudes, values, emotions, interpersonal behaviors, reflective processes, professional dispositions, and patient-centered behaviors.

### Study risk-of-bias assessment

Risk of bias and methodological quality were assessed independently by two reviewers (AZ and NASI) using Joanna Briggs Institute (JBI) critical appraisal tools as the primary appraisal framework (16). The appropriate JBI tool was selected according to study design. The JBI checklist for randomized controlled trials was used for randomized studies, the JBI checklist for quasi-experimental studies was used for non-randomized and pre–post intervention studies, the JBI checklist for analytical cross-sectional studies was used for cross-sectional or validation studies, and the JBI checklist for qualitative research was used for qualitative studies. For mixed-methods studies, the dominant study component most relevant to the review outcome was appraised using the closest corresponding JBI tool. Any discrepancies or uncertain appraisal decisions were discussed and resolved by consensus between the two reviewers.

Each applicable JBI item was rated as “Yes”, “No”, “Unclear”, or “Not applicable”. For scoring purposes, “Yes” was assigned 1 point, while “No” and “Unclear” were assigned 0 points. Items rated “Not applicable” were excluded from the denominator. A percentage score was calculated for each study by dividing the number of “Yes” responses by the total number of applicable items. Methodological quality was categorized as high when ≥70% of applicable items were rated “Yes”, moderate when 50–69% were rated “Yes”, and low when < 50% were rated “Yes”. A minimum methodological quality threshold of 50% was applied for inclusion in the final synthesis.

The Critical Appraisal Skills Programme (CASP) checklist (17) was used only as a supplementary interpretive aid for studies with substantial qualitative or mixed-methods components. CASP was not used for scoring, quality categorization, or inclusion/exclusion decisions. Instead, it helped support interpretive consideration of qualitative reporting, reflexivity, data collection, analysis, and the credibility of qualitative findings during narrative synthesis. The formal quality score, quality category, and inclusion decision for each study were based solely on the JBI appraisal.

All included studies met the minimum JBI methodological quality threshold and were retained in the final synthesis. Methodological limitations were considered during narrative interpretation, and studies with moderate quality or important design limitations were given less interpretive weight than higher-quality comparative, validation, or qualitative studies. The detailed item-level JBI appraisal, including the tool used, item-level ratings, total score, percentage score, quality category, and inclusion decision for each study, is provided in Supplementary Table 2.

### Assessment of reporting bias and missing results

Risk of bias due to missing results was considered narratively by examining whether affective-domain outcomes described in the study aims, methods, intervention descriptions, or assessment tools were reported in the results. The review also considered whether studies showed incomplete reporting of affective-domain measures, selective emphasis on favorable outcomes, absence of protocols or trial registrations, or limited reporting of negative, neutral, or unintended findings. Formal assessment of publication bias using funnel plots or statistical tests was not performed because no meta-analysis was conducted and the included studies were heterogeneous in design, interventions, outcomes, and effect measures.

### Synthesis methods

A narrative synthesis approach was used because of heterogeneity in study designs, learner populations, healthcare disciplines, AI tools, intervention formats, comparators, affective-domain outcomes, measurement instruments, and reporting methods. The included studies were first summarized descriptively according to study characteristics, learner group, AI tool or intervention, educational context, affective-domain focus, and key findings.

Findings were then grouped into thematic categories reflecting the main educational uses of AI and the affective-domain areas addressed by the studies. These categories included communication skills training, breaking bad news and sensitive communication, AI-generated feedback and reflective learning, learner confidence and preparedness, virtual patient and OSCE-based simulation, AI-enhanced virtual reality, and learner or faculty perceptions.

Meta-analysis was not performed because the included studies were not sufficiently homogeneous in terms of design, intervention, comparator, outcome measure, and reporting format. No common effect measure was therefore pre-specified. Where quantitative estimates were reported, including mean differences, change scores, percentages, frequencies, confidence intervals, *p* values, or other study-specific estimates, these were extracted and summarized narratively. Qualitative and mixed-methods findings, including learner perceptions, acceptability, usability, feasibility, engagement, and implementation barriers or facilitators, were synthesized thematically where appropriate.

Sensitivity analyses were not performed because this review did not include meta-analysis or pooled effect estimates. The included studies were highly heterogeneous in terms of study design, learner population, healthcare discipline, AI intervention type, comparator, affective-domain outcome, measurement instrument, and reporting format. Therefore, there was no quantitative synthesis from which sensitivity analyses could be meaningfully derived. Instead, methodological differences and study limitations were considered narratively during synthesis and interpretation.

### Certainty of evidence rating

The certainty of evidence was considered narratively rather than through formal outcome-level GRADE rating. Although the registered protocol specified that GRADE would be used where appropriate, the included studies were too heterogeneous in design, AI intervention type, learner population, comparator, affective-domain outcome, measurement instrument, and reporting format to support pooled quantitative synthesis or formal GRADE summary-of-findings tables. Therefore, formal GRADE assessment was not considered appropriate for this evidence base and was not treated as a major deviation from the protocol. Certainty was instead discussed using GRADE-informed considerations, including risk of bias, inconsistency, indirectness, imprecision, and possible reporting bias. For qualitative and mixed-methods findings, confidence in the evidence was considered in relation to methodological quality, coherence, adequacy, relevance, and consistency of findings.

### Ethical considerations

Ethical approval was obtained from the Research Ethics Committee of The National University of Malaysia. The ethics approval reference number was JEP-2026-092, with an approval period from 9 April 2026 to 8 October 2026. The institutional project code was FF-2026-158, and the project received financial support from the Faculty of Medicine Fundamental Grants. As this review synthesized data from published studies, it did not involve direct participant recruitment, collection of identifiable personal information, or access to unpublished individual-level data.

## Results

### Study selection

The database search identified 1,559 records. After removing 116 duplicates, 1,443 records were screened by title and abstract, and 1,094 were excluded. Of 349 reports sought for retrieval, 83 could not be retrieved. The remaining 266 full-text reports were assessed for eligibility, of which 249 were excluded because they were review articles (*n* = 161), involved non-healthcare student populations (*n* = 85), or did not report an affective-domain outcome (*n* = 3). Seventeen studies met the eligibility criteria and were included in the final synthesis. The study selection process is shown in Figure 1.

### Study characteristics

The 17 included studies examined AI-based educational tools and interventions among undergraduate medical students, post-graduate trainees, residents, pharmacy students, nursing students, and pediatric trainees. The AI tools included LLM-based chatbots, AI-simulated patients, virtual patient platforms, AI-generated feedback systems, avatar-mediated feedback, AI-enhanced virtual reality simulations, and OSCE-based AI assessment platforms.

The included studies mainly addressed AI-supported communication training, simulated patient interaction, reflective feedback, breaking-bad-news communication practice, medical interview rehearsal, OSCE preparation, and learner confidence. The affective-domain outcomes most frequently reported were communication skills, confidence, self-efficacy, reflective learning, professionalism, patient-centered interaction, learner preparedness, and empathy-related learning.

A concise summary of each included study, including learner group, AI tool or intervention, affective-domain focus, and key findings, is presented in Table 1. Detailed study characteristics, including educational context, comparator, key findings, and reported limitations, are provided in Supplementary Table 3.

**Table 1:** Summary of included studies and key affective-domain findings.

References	Study	Year	Country	Learner group/sample	Study design	AI tool or intervention	Affective-domain focus/measure	Key findings
(18)	Bakhaya et al.	2026	Sweden	Pharmacy students and faculty; *n* = 14	Development and qualitative feasibility evaluation	ChatGPT-based chatbot-simulated patients with automated feedback	Written communication, confidence, feedback engagement; semi-structured interviews	Participants perceived the simulated patients as authentic and useful for synchronous written communication practice. Feedback was viewed as structured, detailed, fair, and consistent.
(19)	Jacobs et al.	2025	United Kingdom	Third-year medical students and practicing doctors; *n* = 18, including 15 students and 3 doctors	Mixed-methods sequential explanatory feasibility study	AI virtual patient using LLM and natural voice synthesis	Communication, psychological safety, usability, engagement, fidelity, immersion, debriefing; ITEM-based survey and focus groups	Students reported high intrinsic motivation, usability, psychological safety, and positive learning experience. Participants valued the safe practice environment and immediate feedback.
(20)	Kong et al.	2021	Singapore	Second-year medical students; *n* = 106	Pilot evaluation/short communication	Virtual Integrated Patient with natural language processing and AI-enabled virtual patient functions	History taking, confidence, competence, content retention; user experience survey	Most students reported that VIP helped them remember content; 69% felt it increased confidence and competence in history taking.
(21)	Sheth et al.	2025	Canada	Undergraduate medical students; *n* = 24, generating 93 survey responses and 17 interviews	Multimethod study	OSCEai LLM-based virtual patients	History taking, communication confidence, feedback, anxiety reduction; surveys and interviews	Students reported a 14.6% increase in comfort with history taking. Strengths included flexibility, accessibility, detailed feedback, and a judgement-free practice space.
(22)	Suárez-García et al.	2025	Mexico	Clinical-phase medical students; *n* = 30	Single-arm pre–post simulation study	DIALOGUE, GPT-4o-based AI-simulated patient with automated feedback	Diagnostic communication, empathy-related outcomes, patient-centered consultation; eight-domain communication rubric	Mean total performance increased by 36.7 points; high-performing students increased from 0% to 70%. Gains were significant across all rubric domains.
(23)	Sun et al.	2026	China	Senior clinical medical students; *n* = 82, AI-VSP *n* = 40 and traditional SP *n* = 42	Parallel mixed-methods randomized controlled trial	AI-based virtual standardized patients using DeepSeek-chat and SEGUE framework	Physician–patient communication, patient-centered interaction; SEGUE total, Content, and Process scores; qualitative feedback	Both groups improved, but AI-VSP students achieved higher total and Content scores and completed more training sessions. AI-VSPs were perceived as repeatable, convenient, realistic, intelligent, and helpful.
(24)	Tyrrell et al.	2025	United Kingdom	Undergraduate medical students; *n* = 396 eligible, *n* = 378 completed at least one survey	Multicentre randomized crossover study	SimConverse web-based AI-driven voice-recognition virtual patient simulator	Consultation skills, communication confidence, satisfaction, cost	Both AI-based and actor-based training improved self-reported communication skills. AI-CST was slightly inferior to actor-based training but substantially cheaper per student.
(25)	Yamamoto et al.	2024	Japan	Fourth-year medical students; AI group *n* = 35, historical control *n* = 110	Non-randomised controlled trial	GPT-4 Turbo AI-simulated patients with feedback, delivered via miibo and LINE	Medical interview skills, confidence, OSCE preparation; pre-CC OSCE medical interview score, SBT-QA10	The AI intervention group achieved higher medical interview OSCE scores than controls. The platform was safe and supported repeated practice.
(26)	Armstrong et al.	2026	United Kingdom	Post-graduate medical educators, resident doctors, and doctors across 19 specialties; 69 doctors. Lay representative, *n* = 11, contributed as evaluators/ stakeholders	Quantitative evaluation/anonymous online consultation	ChatGPT-generated breaking-bad-news guidance	Breaking bad news, sensitive communication, professionalism; Likert agreement and preference ratings	AI-generated guidance produced highly rated statements and was preferred by 61% of respondents, compared with 22% preferring the traditional approach.
(27)	Chiu et al.	2025	United States	Second-year medical students; *n* = 12	Pilot pre–post educational intervention	ChatGPT-3.5 simulated patient using SPIKES breaking-bad-news scenario	Confidence, professionalism, trust in AI, difficult communication; pre–post Likert survey and grading comparison	Student confidence in breaking bad news increased significantly, and trust in ChatGPT for communication skills and professionalism training also increased.
(28)	Karnieli-Miller and Elyoseph	2026	Israel	Senior/final-year medical students; *n* = 99	Prospective single-arm pre–post mixed-methods study	BRAVE generative AI-based breaking-bad-news simulator with reflective debrief and SPwICES-mapped feedback	Self-efficacy, willingness, intention to apply protocol, reflective debrief, user experience	Self-efficacy, willingness, and intention to apply the protocol improved significantly. Students perceived BRAVE as useful, realistic, safe, and feedback-rich.
(29)	Haider et al.	2025	Canada	First- and second-year MD students; *n* = 15	Mixed-methods feedback comparison study	ChatGPT-4 feedback on written narrative reflections	Reflective learning, professional identity formation, feedback engagement; Likert ratings and open-ended responses	ChatGPT was rated higher for identifying strengths, areas for improvement, and actionable steps. Students still valued facilitator feedback for relational and personal depth.
(30)	Bauermann et al.	2025	Germany	Medical students; *n* = 82, including 66 second semester and 16 fourth-semester students	Video-based simulation acceptance study	AI-based, avatar-mediated feedback and feedforward	Communication feedback, feedforward, trustworthiness, learning acceptance, avatar perception	AI-based avatar-mediated feedforward was rated more trustworthy and more acceptable for learning than feedback. Avatar sympathy, human-likeness, trustworthiness, and attitudes toward AI influenced acceptance.
(31)	Ba et al.	2024	China	Pediatric trainees/medical interns; *n* = 77, ChatGPT-assisted *n* = 39 and traditional *n* = 38	Controlled experimental study with blinded assessment	ChatGPT-4-assisted teaching	Patient communication, clinical judgement, professional conduct, clinical skills; Mini-CEX and theoretical examination	The ChatGPT-assisted group showed better Mini-CEX performance, especially in patient communication and clinical judgement, while theoretical knowledge scores were similar.
(32)	Bentegeac et al.	2025	France	Undergraduate medical students from five medical schools; *n* = 91	Multicentre prospective validation pilot study	ECOSBot, GPT-4o-powered standardized patient and examiner	OSCE practice, communication, professional attitude, feedback; authenticity, correctness, relevance, ICC, CUQ	ECOSBot showed high fidelity as a standardized patient and strong agreement with human raters for global scores. Most students found the tool useful for OSCE preparation.
(33)	Takahashi et al.	2026	Japan	Mixed clinical experience participants; *n* = 7, including medical students, residents, and attending physicians	Cross-sectional validation study	GPT-o1 Pro and GPT-5 Pro AI-based assessment of interview transcripts in an AI-simulated patient system	Assessment reproducibility, communication evaluation; Master Interview Rating Scale, reliability, agreement, assessment time	AI-based assessment produced scores similar to human assessment, showed strong agreement, higher consistency, and reduced evaluation time by more than half.
(34)	He et al.	2026	United States	Undergraduate nursing students; *n* = 23	Qualitative study	Generative AI-enhanced virtual reality patient encounter simulation	Communication, confidence, motivation, critical thinking, empathy-related learning; individual interviews	Students reported positive learning experiences and perceived the simulation as effective for practicing patient encounters, communication, confidence, and critical thinking.

### Results of individual studies

Table 1 summarizes the key characteristics and affective-domain findings of the 17 included studies, while Supplementary Table 3 provides the detailed extraction table, including educational context, comparator, key findings, and reported limitations. Communication-related outcomes were most frequently reported, including written patient communication, consultation structure, medical interview skills, patient-centered communication, breaking bad news, and OSCE-based communication performance (18–28, 32, 33). Several studies reported improvements in learner confidence, comfort, self-efficacy, preparedness, or willingness to engage in patient communication after AI-supported practice (20, 21, 25, 27, 28, 31, 34). AI-generated feedback was evaluated through automated chatbot feedback, ChatGPT feedback on reflective writing, and avatar-mediated feedback or feedforward (18, 29, 30). AI-supported assessment was examined in GPT-4o-based OSCE simulation and AI-based assessment of medical interview transcripts (32, 33). One study evaluated generative AI-enhanced virtual reality simulation among nursing students and reported improvements in motivation, communication skills, confidence, and critical thinking, although empathy-building was limited by reduced emotional connection with virtual patients (34).

Across studies, reported benefits included repeated low-stakes practice, flexible access, structured feedback, increased learner confidence, improved communication performance, and usefulness for formative preparation (18–25, 27–31, 34). Reported limitations included repetitive or mechanical responses, delayed replies, occasional inaccuracies, overly detailed feedback, limited emotional realism, reduced non-verbal communication practice, difficulty building rapport, and the need for educator oversight (18, 19, 21, 24, 27, 29, 30, 32–34). Overall, AI-based tools were most consistently useful as adjunctive supports for communication rehearsal, feedback, reflection, and confidence-building, while evidence for deeper affective-domain development, including empathy, emotional responsiveness, relational authenticity, and professional identity formation, remained limited (24, 27, 29, 32–34).

### Risk of bias in studies

The detailed item-level JBI quality appraisal is provided in Supplementary Table 2. All 17 included studies met the minimum methodological quality threshold of 50% and were retained in the final synthesis. Based on the pre-defined quality categories, most studies were rated as high methodological quality, while a smaller number were rated as moderate quality. No study was rated as low quality.

Common methodological limitations included small sample sizes, single-center designs, voluntary or convenience sampling, short intervention duration, reliance on self-reported or perception-based outcomes, limited follow-up, and inconsistent use of validated affective-domain measures. At least seven studies included small samples of fewer than 30 participants (18, 19, 21, 27, 29, 33, 34), and several studies used single-arm, pilot, feasibility, qualitative, or perception-based designs (18–22, 27–30, 34). Many studies assessed immediate post-intervention outcomes or learner perceptions rather than long-term behavioral change, workplace performance, patient outcomes, or transfer to real clinical encounters (18–25, 27–34). Controlled, comparative, and validation studies provided relatively stronger evidence for communication performance, OSCE preparation, and assessment reproducibility (23–25, 31–33), whereas qualitative and mixed-methods studies mainly contributed evidence on learner experience, perceived realism, psychological safety, feedback engagement, and implementation concerns (18, 19, 21, 28, 29, 34).

### Synthesis of results

#### AI-supported communication skills training

Communication skills were the most frequently targeted affective-domain outcome. AI was used as chatbot-simulated patients, virtual patients, AI communication trainers, and AI-simulated patient platforms to support written communication, consultation structure, history taking, medical interview rehearsal, patient-centered communication, and OSCE preparation (18–25). Learners valued these tools for repeated practice, flexible access, psychologically safe rehearsal, and automated or structured feedback (18–21, 23–25).

Across communication-focused studies, AI appeared most useful as an adjunct to conventional teaching rather than a replacement for human-led communication training. AI tools supported independent rehearsal and helped learners practice the structure and content of clinical conversations (20, 21, 23, 25). However, learners and faculty reported repetitive responses, delayed replies, mechanical tone, occasional inaccuracies, overly detailed answers, and reduced rapport-building compared with human standardized patients (19, 21, 24). Text-based or mechanically voiced systems also limited practice in non-verbal communication, emotional responsiveness, and embodied interpersonal behaviors (21, 24).

#### Breaking bad news and sensitive communication

Three studies focused on breaking bad news and emotionally sensitive communication (26–28). AI-generated guidance and simulation helped learners prepare the structure, wording, and sequence of difficult conversations (26–28). ChatGPT-based SPIKES training and generative AI-based breaking-bad-news simulation were associated with improved confidence, self-efficacy, willingness, trust, and intention to apply structured communication protocols (27, 28).

However, AI systems remained limited in reproducing emotional expression, vocal tone, body language, non-verbal cues, distress, silence, and uncertainty (27, 28). This is important because sensitive communication requires not only appropriate wording but also responsiveness to patients' emotional reactions and relational needs.

#### AI-generated feedback, feedforward, and reflective learning

AI-generated feedback was used for communication training, reflective writing, and consultation-based learning (18, 29, 30). Feedback was valued as immediate, structured, detailed, consistent, and actionable (18, 29, 30). In reflective writing, ChatGPT-generated feedback helped identify strengths, areas for improvement, and suggestions for deeper reflection (29). In communication training, avatar-mediated feedback and feedforward were perceived as acceptable and trustworthy, with feedforward supporting future improvement (30).

However, AI-generated feedback was limited by generic wording, repetition, excessive length, occasional inaccuracies, and limited capacity to interpret emotional meaning, contextual nuance, or professional identity formation (18, 29, 30). Learners continued to value facilitator feedback because educators could provide personal, relational, and contextual understanding that AI could not fully reproduce (29). AI feedback may therefore be useful as formative support, but educator oversight remains important when feedback involves professionalism, empathy, reflection, or complex interpersonal judgement (29, 30).

#### Clinical confidence, professionalism, and learner preparedness

Several studies reported improvements in learner confidence, comfort, self-efficacy, preparedness, or professional performance after AI-supported learning (20, 21, 25, 27, 28, 31). ChatGPT-assisted pediatric teaching was associated with better Mini-CEX performance in clinical judgement, doctor–patient communication, professional conduct, and overall clinical skills, despite similar theoretical knowledge scores compared with traditional teaching (31). Other studies reported improved confidence, comfort, willingness, or preparedness after AI-supported history-taking and breaking-bad-news practice (20, 21, 27, 28).

However, most confidence and preparedness outcomes were short-term and perception-based (20, 21, 27, 28). Evidence for sustained behavioral change, transfer to real patient care, or long-term professional development remained limited.

#### AI in virtual patient and OSCE-based simulation

AI was used in OSCE-based simulation and assessment, including standardized patient simulation, standardized examiner simulation, and transcript-based medical interview assessment (25, 32, 33). These tools showed potential for structured practice, formative scoring, and faculty-verified assessment workflows (32, 33). GPT-4o-powered OSCE simulation produced clinically relevant and contextually appropriate standardized patient responses, while AI-based transcript assessment produced ratings comparable to human assessment and showed favorable psychometric properties (32, 33).

However, AI assessment was weaker in domains requiring interpretation of communication quality, professional attitude, interview technique, empathy, and interpersonal nuance (32, 33). Transcript-based assessment may also miss non-verbal and contextual elements central to affective-domain assessment (33). AI may therefore be better suited for structured formative scoring or preliminary feedback than for independent high-stakes assessment of affective-domain competencies (32, 33).

#### AI-enhanced virtual reality and empathy-related learning

One study examined generative AI-enhanced virtual reality patient encounter simulation among nursing students (34). Students reported improved motivation, communication skills, confidence, critical thinking, and ability to manage unexpected patient responses (34). Generative AI allowed virtual patients to demonstrate varied personalities and conversational styles, creating a more adaptive and interactive simulation experience (34).

However, empathy-related learning remained limited. Students found it difficult to establish emotional connection with virtual patients because AI-generated voices were perceived as robotic or synthetic (34). Similar limitations were reported in other studies where AI supported structured communication practice but struggled to reproduce emotional nuance, natural voice, body language, facial expression, and relational presence (24, 27, 34).

### Overall pattern of findings

Across the included studies, AI-based tools appeared most useful for communication rehearsal, structured feedback, reflective learning, learner confidence, and formative assessment preparation (18–34). Their strongest contribution was providing repeated low-stakes practice, immediate feedback, flexible access, and scalable support for communication-related learning (18–25, 29–34).

Evidence was less convincing for deeper affective-domain outcomes such as empathy, emotional responsiveness, relational authenticity, professional identity formation, and non-verbal communication (24, 27, 29, 32–34). Most studies evaluated short-term outcomes, learner perceptions, or simulation-based performance rather than sustained behavioral change in real clinical settings (18–34). Overall, AI-supported tools were best supported as adjunctive tools for communication rehearsal, feedback, confidence-building, and reflective learning, while evidence for deeper affective-domain development remained limited.

## Discussion

### Principal findings

This systematic review synthesized evidence from 17 studies examining AI-supported educational approaches for affective-domain development in health professions education. Across the included studies, AI-supported tools were mainly used as virtual patients, chatbots, simulated patients, automated feedback systems, avatar-mediated feedback tools, OSCE-based platforms, and AI-enhanced virtual reality simulations. These tools were applied to history taking, consultation practice, breaking-bad-news training, reflective writing, communication feedback, and formative assessment preparation (18–34).

The strongest findings were observed where AI supported structured, repeatable, and psychologically safe learning activities. AI-based virtual patients and chatbots allowed learners to rehearse patient interaction, practice consultation structure, and receive feedback in low-stakes settings before engaging with human patients or standardized patients (19–25, 27, 28). Several studies reported improvements in learner confidence, comfort, self-efficacy, preparedness, communication performance, reflective engagement, or perceived usefulness after AI-supported practice (18, 20, 21, 25, 27–31, 34). These findings suggest that AI has practical value as a scalable adjunct for repeated communication rehearsal and formative learning.

However, evidence for deeper affective-domain development was less convincing. Although AI tools supported the structure, wording, sequencing, and feedback processes of communication training, their ability to develop empathy, emotional responsiveness, relational authenticity, professional identity formation, and non-verbal communication remained limited (24, 27, 29, 32–34). This distinction is important because affective-domain development extends beyond communication technique to broader human capabilities, personal resilience, self-awareness, and professional formation (35). Many studies focused on short-term outcomes, learner perceptions, self-reported confidence, or simulation-based performance rather than sustained behavioral change in clinical settings. Therefore, while AI-supported tools may improve access to practice and feedback, current evidence does not show that they can independently develop the full range of affective-domain competencies required for compassionate, patient-centered care.

### Comparison with prior work

These findings extend previous reviews of AI-supported communication education by focusing specifically on affective-domain outcomes rather than primarily on technical performance, knowledge acquisition, or general implementation. Consistent with prior work on virtual patients, AI-supported simulation, and automated feedback, the present review found that AI was most useful for structured practice, repeated exposure, immediate feedback, and psychologically safe rehearsal. Viewed through Krathwohl's affective-domain framework, these functions may support earlier levels of affective learning, particularly receiving, responding, and in some cases valuing. However, evidence that AI independently supports higher levels of affective development, such as organization or internalization of professional values, remains limited.

A recurring theme was the tension between educational usefulness and simulation realism. AI-supported virtual patients, chatbots, and conversational systems were useful for practicing history taking, consultation flow, diagnostic communication, and breaking bad news (19, 21–23, 27, 28). However, learners and educators reported limitations in emotional realism, dynamic response, rapport-building, vocal tone, body language, non-verbal cues, and embodied interpersonal presence (21, 24, 27, 34). This matters because affective-domain education depends not only on appropriate wording, but also on recognizing and responding to silence, hesitation, distress, uncertainty, discomfort, and subtle emotional cues, which are central to empathy, patient-centered communication, and relational competence (8, 10, 35). AI may therefore help learners practice the structure of empathic communication, but it remains insufficient as a standalone replacement for human standardized patients, actor-based simulation, educator-led debriefing, and authentic clinical encounters.

AI also contributed to feedback and feedforward. AI-generated feedback was valued for being immediate, structured, detailed, consistent, and accessible (18, 29, 30). In reflective writing, ChatGPT-generated feedback helped learners identify strengths, areas for improvement, and actionable steps for deeper reflection (29). In communication training, avatar-mediated feedback and feedforward were perceived as acceptable and trustworthy, with feedforward supporting future improvement (30). These findings are relevant because affective-domain development requires not only evaluation of past performance, but also guidance for future behavioral and reflective growth.

Despite these benefits, AI-generated feedback should not replace educator judgement. Concerns remained regarding generic wording, excessive length, repetition, occasional inaccuracies, and limited ability to interpret emotional meaning, professional identity formation, or contextual nuance (18, 29, 30). Students continued to value facilitator feedback because human educators could provide personal, relational, and contextual understanding that AI could not fully reproduce (29). AI-generated feedback may therefore be most appropriate as formative support, with educator oversight retained for empathy, professionalism, reflection, ethical reasoning, and complex interpersonal judgement.

This review also identified emerging potential for AI-supported assessment. AI tools used in OSCE and medical interview contexts showed promise for structured practice, preliminary scoring, transcript-based evaluation, and faculty-verified assessment workflows (25, 32, 33). However, AI assessment appeared stronger for structured content, clinical reasoning, global scoring, and reproducibility than for domains requiring interpretation of empathy, professional attitude, communication quality, and interpersonal nuance (32, 33). These findings align with concerns that generative AI requires educators to rethink the value, purpose, and validity of assessment rather than simply automate existing assessment practices (36). AI may therefore be useful in an AI-first, faculty-verified workflow, where AI provides preliminary scoring or feedback, while faculty retain responsibility for final judgement, contextual interpretation, and high-stakes decisions.

The findings also reinforce the need for responsible curriculum integration. Learning technologies in health professions education should be adopted not because they are novel or efficient, but because they meaningfully support educational goals, learner development, and professional practice (37). AI-supported affective-domain training should be mapped to explicit outcomes, integrated with human facilitation, and aligned with AI competency frameworks that emphasize responsible use, communication, collaboration, ethics, safety, and quality improvement (38, 39). Implementation should also address ethical and equity considerations, including transparency, bias, accountability, privacy, and the risk that AI systems may reproduce or amplify inequities in educational practice (40, 41).

Overall, the findings support AI as a complementary educational tool rather than a replacement for human-facilitated affective-domain education. AI may be most useful before face-to-face teaching to prepare learners, between simulation sessions to support repeated rehearsal, and after clinical or simulated encounters to support reflection and feedback (21, 28, 29). However, educator guidance, human debriefing, and authentic clinical interaction remain essential for developing empathy, relational responsiveness, ethical judgement, and professional identity (35). Future AI-supported affective-domain training should therefore be integrated within blended curricula and mapped to outcomes such as communication, empathy, professionalism, self-awareness, ethical responsibility, and patient-centered care.

### Limitations

This review has several limitations. First, the included studies were heterogeneous in learner population, healthcare discipline, AI tool, educational context, study design, outcome measure, and intervention duration. This limited direct comparison and precluded meta-analysis. Therefore, findings were synthesized narratively, and conclusions should be interpreted cautiously.

Although all included studies met the minimum JBI methodological quality threshold for inclusion, the evidence base remained limited by the pre-dominance of small, pilot, feasibility, single-arm, and perception-based studies. Therefore, the quality appraisal informed the weighting and interpretation of findings rather than leading to exclusion of any included study.

Second, many studies used small samples, single-center designs, voluntary participation, short intervention periods, and immediate post-intervention assessment. These features limit generalisability and make it difficult to determine whether improvements in confidence, self-efficacy, communication performance, or learner preparedness are sustained over time.

Third, many outcomes were self-reported or perception-based. Although learner perceptions are important for evaluating acceptability, usability, and psychological safety, they do not necessarily reflect behavioral change, transfer to patient care, or deeper affective-domain development. Few studies assessed long-term outcomes, workplace performance, patient perspectives, or objective measures of empathy, professionalism, reflective capacity, or patient-centered behavior.

Fourth, affective-domain outcomes were inconsistently conceptualized and measured. Communication skills, empathy, professionalism, confidence, self-efficacy, reflection, and patient-centered interaction were assessed using different tools, study-specific questionnaires, or qualitative feedback. This variability limited comparison across studies and made it difficult to determine which AI approaches were most effective for specific affective-domain competencies.

Fifth, the review was limited to English-language published studies with accessible full texts. Relevant non-English studies, unpublished studies, trial registrations, institutional reports, theses, pre-prints, or conference abstracts without full papers may have been missed. This may limit cultural generalisability, particularly because communication, empathy, professionalism, and patient-centered interaction are shaped by language, culture, health literacy, and local expectations of clinician–patient relationships. In addition, rapidly evolving terminology around AI, generative AI, chatbots, virtual patients, and LLM-based simulation may have led to missed studies despite broad search terms. Exclusion of gray literature may also have introduced publication bias because studies with positive or favorable findings on AI-supported educational tools may be more likely to appear in peer-reviewed journals, while neutral, incomplete, unsuccessful, or negative implementations may be underrepresented. As a result, this review may overestimate the educational usefulness and acceptability of AI-supported affective-domain interventions.

Sixth, reporting bias could not be fully excluded. Not all included studies provided protocols, trial registrations, or sufficiently detailed reporting of planned outcomes, and some studies relied on perception-based or selectively emphasized favorable findings. Although risk of bias due to missing results was considered narratively, formal assessment of publication bias using funnel plots or statistical tests was not possible because no meta-analysis was conducted and the included studies were heterogeneous.

Finally, although this review focused on AI-supported affective-domain development, some included studies also reported cognitive, technical, or clinical performance outcomes. Distinguishing affective-domain learning from broader educational outcomes was sometimes challenging, especially when studies reported communication performance, clinical judgement, or learner confidence alongside cognitive or technical measures. In addition, although the registered protocol stated that GRADE would be applied where appropriate, formal outcome-level GRADE assessment was not undertaken because the evidence was not suitable for pooled outcome-level certainty grading or summary-of-findings tables. Sensitivity analyses were also not performed because there were no pooled effect estimates to test. Therefore, the findings should be interpreted as a narrative, GRADE-informed synthesis of emerging evidence rather than as formal certainty-rated or quantitatively pooled evidence.

## Conclusions

This systematic review suggests that AI-based educational tools can support selected aspects of affective-domain development in health professions education, particularly communication rehearsal, structured feedback, reflective learning, learner confidence, preparedness for patient interaction, and formative assessment preparation. AI-supported virtual patients, chatbots, automated feedback systems, OSCE tools, and AI-enhanced simulations appear most useful as scalable, flexible, and psychologically safe adjuncts to existing teaching.

However, current evidence remains limited for deeper affective-domain outcomes such as empathy, emotional responsiveness, relational authenticity, professional identity formation, and non-verbal communication. AI can help learners practice the structure and language of clinical communication, but it should complement rather than replace educator-led teaching, human standardized patients, reflective debriefing, and authentic clinical experience. Future research should use larger, multicentre, longitudinal, and comparative designs with validated affective-domain measures and objective performance assessments to determine whether AI-supported training leads to sustained behavioral change and patient-centered outcomes.

## Data Availability

The original contributions presented in the study are included in the article/Supplementary material, further inquiries can be directed to the corresponding author.
