# Supplementary material for: Artificial intelligence for affective-domain development in healthcare professions education: a systematic review
Source: Front Med (Lausanne). 2026 Jul 30;13:1894820. doi: 10.3389/fmed.2026.1894820 (PMC13470254; doi:10.3389/fmed.2026.1894820)
Supplement: Supplementary file 2 [file Table_1.docx]

**Supplementary Table 1.** Full database-specific search strategies

| Database / source | Date searched | Search strategy / search equation | Field codes / search fields | Limits applied |
| --- | --- | --- | --- | --- |
| PubMed | 7 May 2026 | (“Artificial Intelligence”[MeSH] OR “Machine Learning”[MeSH] OR “Natural Language Processing”[MeSH] OR “artificial intelligence”[Title/Abstract] OR “generative AI”[Title/Abstract] OR “GenAI”[Title/Abstract] OR ChatGPT[Title/Abstract] OR chatbot*[Title/Abstract] OR “large language model”[Title/Abstract] OR LLM[Title/Abstract] OR “virtual patient”[Title/Abstract] OR “simulated patient”[Title/Abstract] OR “virtual standardized patient”[Title/Abstract] OR “virtual standardised patient”[Title/Abstract] OR “automated feedback”[Title/Abstract] OR “AI feedback”[Title/Abstract]) AND (“Education, Medical”[MeSH] OR “Education, Nursing”[MeSH] OR “Education, Pharmacy”[MeSH] OR “Students, Medical”[MeSH] OR “Students, Nursing”[MeSH] OR “Students, Pharmacy”[MeSH] OR “health professions education”[Title/Abstract] OR “healthcare professions education”[Title/Abstract] OR “medical education”[Title/Abstract] OR “nursing education”[Title/Abstract] OR “pharmacy education”[Title/Abstract] OR “dental education”[Title/Abstract] OR “allied health education”[Title/Abstract] OR “medical student”[Title/Abstract] OR “nursing student”[Title/Abstract] OR “pharmacy student”[Title/Abstract] OR trainee*[Title/Abstract] OR resident*[Title/Abstract]) AND (“Communication”[MeSH] OR “Empathy”[MeSH] OR “Professionalism”[Title/Abstract] OR “Patient-Centered Care”[MeSH] OR “Self Efficacy”[MeSH] OR “communication skills”[Title/Abstract] OR empath*[Title/Abstract] OR professionalism[Title/Abstract] OR reflection[Title/Abstract] OR reflective[Title/Abstract] OR confidence[Title/Abstract] OR “self-efficacy”[Title/Abstract] OR “patient-centred care”[Title/Abstract] OR “patient-centered care”[Title/Abstract] OR “breaking bad news”[Title/Abstract] OR “clinical communication”[Title/Abstract] OR “affective learning”[Title/Abstract] OR “affective domain”[Title/Abstract] OR attitude*[Title/Abstract] OR “professional identity”[Title/Abstract]) | MeSH; Title/Abstract | English language; January 2010 to 7 May 2026; empirical studies; human learners |
| Scopus | 7 May 2026 | TITLE-ABS-KEY (“artificial intelligence” OR “generative AI” OR GenAI OR ChatGPT OR chatbot* OR “large language model*” OR LLM OR “virtual patient*” OR “simulated patient*” OR “virtual standardized patient*” OR “virtual standardised patient*” OR “automated feedback” OR “AI feedback”) AND TITLE-ABS-KEY (“health professions education” OR “healthcare professions education” OR “medical education” OR “nursing education” OR “pharmacy education” OR “dental education” OR “allied health education” OR “medical student*” OR “nursing student*” OR “pharmacy student*” OR trainee* OR resident*) AND TITLE-ABS-KEY (“communication skills” OR communication OR empath* OR professionalism OR reflection OR reflective OR confidence OR “self-efficacy” OR “patient-centred care” OR “patient-centered care” OR “breaking bad news” OR “clinical communication” OR “affective learning” OR “affective domain” OR attitude* OR “professional identity”) | TITLE-ABS-KEY | English language; 2010–2026; articles and conference papers screened for empirical eligibility |
| Web of Science Core Collection | 7 May 2026 | TS=(“artificial intelligence” OR “generative AI” OR GenAI OR ChatGPT OR chatbot* OR “large language model*” OR LLM OR “virtual patient*” OR “simulated patient*” OR “virtual standardized patient*” OR “virtual standardised patient*” OR “automated feedback” OR “AI feedback”) AND TS=(“health professions education” OR “healthcare professions education” OR “medical education” OR “nursing education” OR “pharmacy education” OR “dental education” OR “allied health education” OR “medical student*” OR “nursing student*” OR “pharmacy student*” OR trainee* OR resident*) AND TS=(“communication skills” OR communication OR empath* OR professionalism OR reflection OR reflective OR confidence OR “self-efficacy” OR “patient-centred care” OR “patient-centered care” OR “breaking bad news” OR “clinical communication” OR “affective learning” OR “affective domain” OR attitude* OR “professional identity”) | Topic search: title, abstract, author keywords, Keywords Plus | English language; 2010–2026; articles, early access articles, and proceedings screened for empirical eligibility |
| SpringerLink | 7 May 2026 | (“artificial intelligence” OR “generative AI” OR ChatGPT OR chatbot OR “large language model” OR LLM OR “virtual patient” OR “simulated patient” OR “automated feedback”) AND (“medical education” OR “nursing education” OR “pharmacy education” OR “health professions education” OR “medical students” OR trainees OR residents) AND (“communication skills” OR empathy OR professionalism OR reflection OR confidence OR “self-efficacy” OR “patient-centred care” OR “breaking bad news” OR “affective domain”) | Full-text platform search with title/abstract screening performed after retrieval | English language; 2010–2026; research articles and relevant book chapters screened for empirical eligibility |
| ScienceDirect | 7 May 2026 | (“artificial intelligence” OR “generative AI” OR ChatGPT OR chatbot OR “large language model” OR LLM OR “virtual patient” OR “simulated patient” OR “automated feedback”) AND (“medical education” OR “nursing education” OR “pharmacy education” OR “health professions education” OR “medical students” OR trainees OR residents) AND (“communication skills” OR empathy OR professionalism OR reflection OR confidence OR “self-efficacy” OR “patient-centred care” OR “breaking bad news” OR “affective domain”) | Title, abstract, and keywords where available; platform search refined by subject relevance | English language; 2010–2026; research articles screened for empirical eligibility |
| Wiley Online Library | 7 May 2026 | (“artificial intelligence” OR “generative AI” OR ChatGPT OR chatbot OR “large language model” OR LLM OR “virtual patient” OR “simulated patient” OR “automated feedback”) AND (“medical education” OR “nursing education” OR “pharmacy education” OR “health professions education” OR “medical students” OR trainees OR residents) AND (“communication skills” OR empathy OR professionalism OR reflection OR confidence OR “self-efficacy” OR “patient-centred care” OR “breaking bad news” OR “affective domain”) | Full-text platform search with title/abstract screening performed after retrieval | English language; 2010–2026; journal articles screened for empirical eligibility |
| IEEE Xplore | 7 May 2026 | (“artificial intelligence” OR “generative AI” OR ChatGPT OR chatbot OR “large language model” OR LLM OR “virtual patient” OR “simulated patient” OR “automated feedback” OR “AI feedback”) AND (“medical education” OR “nursing education” OR “pharmacy education” OR “health professions education” OR “medical students” OR trainees OR residents) AND (“communication skills” OR empathy OR professionalism OR reflection OR confidence OR “self-efficacy” OR “patient-centred care” OR “breaking bad news” OR “affective domain”) | All metadata / abstract search where available | English language; 2010–2026; journal articles and conference papers screened for empirical eligibility |
| Google Scholar | 7 May 2026 | Search 1: “artificial intelligence” “medical education” “communication skills” “empathy” Search 2: ChatGPT “medical students” “communication skills” Search 3: “large language model” “virtual patient” “medical education” Search 4: “generative AI” “health professions education” “affective domain” Search 5: “AI feedback” “medical education” “professionalism” OR “reflection” | Google Scholar phrase searching; first relevant result pages screened by title and snippet, followed by full-text eligibility assessment | English language; 2010–2026; empirical studies only; reviews, editorials, commentaries, and non-healthcare learner populations excluded |
| Additional searching | 7 May 2026 | Reference lists of included studies and relevant reviews were screened manually to identify additional eligible empirical studies. | Citation and reference list screening | English language; 2010–2026; full text accessible; empirical studies only |
