# Supplementary material for: Artificial intelligence for affective-domain development in healthcare professions education: a systematic review
Source: Front Med (Lausanne). 2026 Jul 30;13:1894820. doi: 10.3389/fmed.2026.1894820 (PMC13470254; doi:10.3389/fmed.2026.1894820)
Supplement: Supplementary file 3 [file Table_2.docx]

**Supplementary Table 2.** Item-level JBI critical appraisal of included studies.

| **Ref** | **Study** | **Year** | **Study design** | **JBI tool used** | **Item 1** | **Item 2** | **Item 3** | **Item 4** | **Item 5** | **Item 6** | **Item 7** | **Item 8** | **Item 9** | **Item 10** | **Item 11** | **Total Yes** | **Appli-cable items** | **% score** | **Quality category** | **Inclusion decision** |
| --- | --- | --- | --- | --- | --- | --- | --- | --- | --- | --- | --- | --- | --- | --- | --- | --- | --- | --- | --- | --- |
| [18] | Bakhaya et al. | 2026 | Development and qualitative feasibility evaluation | JBI Qualitative Research | Un-clear | Yes | Yes | Yes | Yes | Yes | Un-clear | Yes | Yes | Yes | N/A | 8 | 10 | 80.00% | High | Include |
| [19] | Jacobs et al. | 2025 | Mixed-methods sequential explanatory feasibility study | JBI Quasi-Experimental Studies | Yes | N/A | N/A | No | No | Yes | Yes | Yes | Yes | N/A | N/A | 5 | 7 | 71.40% | High | Include |
| [20] | Kong et al. | 2021 | Pilot evaluation / short communication | JBI Quasi-Experimental Studies | Yes | N/A | N/A | No | No | Yes | Yes | Un-clear | Yes | N/A | N/A | 4 | 7 | 57.10% | Moderate | Include |
| [21] | Sheth et al. | 2025 | Multimethod study | JBI Quasi-Experimental Studies | Yes | N/A | N/A | No | Yes | Yes | Yes | Un-clear | Yes | N/A | N/A | 5 | 7 | 71.40% | High | Include |
| [22] | Suárez-García et al. | 2025 | Single-arm pre–post simulation study | JBI Quasi-Experimental Studies | Yes | N/A | N/A | No | Yes | Yes | Yes | Yes | Yes | N/A | N/A | 6 | 7 | 85.70% | High | Include |
| [23] | Sun et al. | 2026 | Parallel mixed-methods randomised controlled trial | JBI Randomised Controlled Trials | Yes | Un-clear | Yes | No | No | Un-clear | Yes | Yes | Yes | Yes | Yes | 7 | 11 | 63.60% | Moderate | Include |
| [24] | Tyrrell et al. | 2025 | Multicentre randomised crossover study | JBI Randomised Controlled Trials | Yes | Yes | Yes | No | No | No | Yes | Yes | Yes | Un-clear | Yes | 7 | 11 | 63.60% | Moderate | Include |
| [25] | Yamamoto et al. | 2024 | Non-randomised controlled trial / historical control study | JBI Quasi-Experimental Studies | Yes | Yes | Un-clear | Yes | No | Yes | Yes | Yes | Yes | N/A | N/A | 7 | 9 | 77.80% | High | Include |
| [26] | Armstrong et al. | 2026 | Quantitative evaluation / anonymous online consultation | JBI Analytical Cross-Sectional Studies | Yes | Yes | Yes | Yes | N/A | No | Yes | Yes | N/A | N/A | N/A | 6 | 8 | 75.00% | High | Include |
| [27] | Chiu et al. | 2025 | Pilot pre–post educational intervention | JBI Quasi-Experimental Studies | Yes | N/A | N/A | No | Yes | Yes | Yes | Un-clear | Yes | N/A | N/A | 5 | 7 | 71.40% | High | Include |
| [28] | Karnieli-Miller and Elyoseph | 2026 | Prospective single-arm pre–post mixed-methods study | JBI Quasi-Experimental Studies | Yes | N/A | N/A | No | Yes | Yes | Yes | Un-clear | Yes | N/A | N/A | 5 | 7 | 71.40% | High | Include |
| [29] | Haider et al. | 2025 | Mixed-methods feedback comparison study | JBI Analytical Cross-Sectional Studies | Yes | Yes | Yes | Yes | N/A | No | Yes | Yes | N/A | N/A | N/A | 6 | 8 | 75.00% | High | Include |
| [30] | Bauermann et al. | 2025 | Video-based simulation acceptance study | JBI Analytical Cross-Sectional Studies | Yes | Yes | Yes | Yes | N/A | Yes | Yes | Yes | N/A | N/A | N/A | 7 | 8 | 87.50% | High | Include |
| [31] | Ba et al. | 2024 | Controlled experimental study with blinded assessment | JBI Randomised Controlled Trials | Yes | Un-clear | Yes | No | No | Yes | Yes | Yes | Yes | Yes | Yes | 8 | 11 | 72.70% | High | Include |
| [32] | Bentegeac et al. | 2025 | Multicentre prospective validation pilot study | JBI Analytical Cross-Sectional Studies | Yes | Yes | Yes | Yes | Yes | Yes | Yes | Yes | N/A | N/A | N/A | 8 | 8 | 100.00% | High | Include |
| [33] | Takahashi et al. | 2026 | Cross-sectional validation study | JBI Analytical Cross-Sectional Studies | Un-clear | Yes | Yes | Yes | Yes | No | Yes | Yes | N/A | N/A | N/A | 6 | 8 | 75.00% | High | Include |
| [34] | He et al. | 2026 | Qualitative study | JBI Qualitative Research | Un-clear | Yes | Yes | Yes | Yes | Yes | Un-clear | Yes | Yes | Yes | N/A | 8 | 10 | 80.00% | High | Include |
