# Supplementary material for: Artificial intelligence for affective-domain development in healthcare professions education: a systematic review
Source: Front Med (Lausanne). 2026 Jul 30;13:1894820. doi: 10.3389/fmed.2026.1894820 (PMC13470254; doi:10.3389/fmed.2026.1894820)
Supplement: Supplementary file 4 [file Table_3.docx]

**Supplementary Table 3.** Detailed study characteristics, educational context, comparators, key findings, and reported limitations of included studies

| Ref. | Study | Year | Country | Learner group / sample | Study design | AI tool / intervention | Educational context | Affective-domain focus / measure | Comparator | Key findings | Reported limitations |
| --- | --- | --- | --- | --- | --- | --- | --- | --- | --- | --- | --- |
| [18] | Bakhaya et al. | 2026 | Sweden | Pharmacy students and faculty; n = 14 | Development and qualitative feasibility evaluation | ChatGPT-based chatbot-simulated patients with automated feedback | Self-care written communication training in pharmacy education | Written communication, confidence, feedback engagement; semi-structured interviews | None | Participants perceived the simulated patients as authentic and useful for synchronous written communication practice. Feedback was viewed as structured, detailed, fair, and consistent. | Feedback was sometimes lengthy, repetitive, or inaccurate. Findings were based on a small feasibility sample and mainly perception-based outcomes. |
| [19] | Jacobs et al. | 2025 | United Kingdom | Third-year medical students and practising doctors; n = 18, including 15 students and 3 doctors | Mixed-methods sequential explanatory feasibility study | AI virtual patient using LLM and natural voice synthesis | Primary care consultation for prostate cancer risk assessment | Communication, psychological safety, usability, engagement, fidelity, immersion, debriefing; ITEM-based survey and focus groups | None | Students reported high intrinsic motivation, usability, psychological safety, and positive learning experience. Participants valued the safe practice environment and immediate feedback. | Fidelity and immersion were only moderate. Technical limitations and realism constraints were reported; the AI virtual patient did not yet match human standardised patient authenticity. |
| [20] | Kong et al. | 2021 | Singapore | Second-year medical students; n = 106 | Pilot evaluation / short communication | Virtual Integrated Patient with natural language processing and AI-enabled virtual patient functions | Communication with Patients module; history-taking supplement alongside standardised patient tutorials | History taking, confidence, competence, content retention; user experience survey | Usual face-to-face standardised patient tutorials as curricular context, but no direct comparative analysis | Most students reported that VIP helped them remember content; 69% felt it increased confidence and competence in history taking. | Survey-based pilot study; no objective performance assessment or long-term follow-up. |
| [21] | Sheth et al. | 2025 | Canada | Undergraduate medical students; n = 24, generating 93 survey responses and 17 interviews | Multimethod study | OSCEai LLM-based virtual patients | History-taking practice using cases aligned with problem-based learning curriculum | History taking, communication confidence, feedback, anxiety reduction; surveys and interviews | None | Students reported a 14.6% increase in comfort with history taking. Strengths included flexibility, accessibility, detailed feedback, and a judgement-free practice space. | Lower fidelity than standardised patients; feedback was sometimes repetitive or less clinically relevant than preceptor feedback. Evidence was mainly self-reported. |
| [22] | Suárez-García et al. | 2025 | Mexico | Clinical-phase medical students; n = 30 | Single-arm pre–post simulation study | DIALOGUE, GPT-4o-based AI-simulated patient with automated feedback | Type 2 diabetes diagnostic communication training | Diagnostic communication, empathy-related outcomes, patient-centred consultation; eight-domain communication rubric | No parallel comparator; pre-test AI encounters and post-test human standardised patient consultations | Mean total performance increased by 36.7 points; high-performing students increased from 0% to 70%. Gains were significant across all rubric domains. | Single-arm design, small sample, short follow-up, and possible influence of digital self-efficacy. Long-term transfer to real clinical encounters was not established. |
| [23] | Sun et al. | 2026 | China | Senior clinical medical students; n = 82, AI-VSP n = 40 and traditional SP n = 42 | Parallel mixed-methods randomised controlled trial | AI-based virtual standardised patients using DeepSeek-chat and SEGUE framework | Physician–patient communication training after medical ethics course | Physician–patient communication, patient-centred interaction; SEGUE total, Content, and Process scores; qualitative feedback | Traditional human standardised patients | Both groups improved, but AI-VSP students achieved higher total and Content scores and completed more training sessions. AI-VSPs were perceived as repeatable, convenient, realistic, intelligent, and helpful. | Process scores were similar between groups, and AI-VSPs did not necessarily improve empathy or relational communication. |
| [24] | Tyrrell et al. | 2025 | United Kingdom | Undergraduate medical students; n = 396 eligible, n = 378 completed at least one survey | Multicentre randomised crossover study | SimConverse web-based AI-driven voice-recognition virtual patient simulator | Primary care consultation skills training | Consultation skills, communication confidence, satisfaction, cost | Actor-based consultation skills training | Both AI-based and actor-based training improved self-reported communication skills. AI-CST was slightly inferior to actor-based training but substantially cheaper per student. | Outcomes were self-reported, satisfaction was lower for AI-CST, and the intervention was a single-session simulation rather than long-term clinical assessment. |
| [25] | Yamamoto et al. | 2024 | Japan | Fourth-year medical students; AI group n = 35, historical control n = 110 | Non-randomised controlled trial | GPT-4 Turbo AI-simulated patients with feedback, delivered via miibo and LINE | Medical interview practice before pre-clinical clerkship OSCE | Medical interview skills, confidence, OSCE preparation; pre-CC OSCE medical interview score, SBT-QA10 | Traditional training alone / historical control | The AI intervention group achieved higher medical interview OSCE scores than controls. The platform was safe and supported repeated practice. | Non-randomised historical comparison; short-term OSCE-based assessment only; limited effect on non-verbal communication; should supplement rather than replace traditional training. |
| [26] | Armstrong et al. | 2026 | United Kingdom | Postgraduate medical educators, resident doctors, and doctors across 19 specialties; 69 doctors. Lay representatives, n = 11, contributed as evaluators/stakeholders rather than as the primary learner population. | Quantitative evaluation / anonymous online consultation | ChatGPT-generated breaking-bad-news guidance | Generic Professional Capabilities and postgraduate breaking-bad-news guidance | Breaking bad news, sensitive communication, professionalism; Likert agreement and preference ratings | Human-generated guidance from facilitated trainer–resident discussions | AI-generated guidance produced highly rated statements and was preferred by 61% of respondents, compared with 22% preferring the traditional approach. | Did not assess learner performance or real communication behaviour. Small numbers limited subgroup generalisability; AI content required appropriate curation. |
| [27] | Chiu et al. | 2025 | United States | Second-year medical students; n = 12 | Pilot pre–post educational intervention | ChatGPT-3.5 simulated patient using SPIKES breaking-bad-news scenario | Breaking-bad-news communication training | Confidence, professionalism, trust in AI, difficult communication; pre–post Likert survey and grading comparison | No intervention comparator; ChatGPT and faculty grading compared | Student confidence in breaking bad news increased significantly, and trust in ChatGPT for communication skills and professionalism training also increased. | Small sample; ChatGPT grading was inconsistent and differed from faculty ratings. Emotional realism, voice tone, body language, and non-verbal cues were limited. |
| [28] | Karnieli-Miller and Elyoseph | 2026 | Israel | Senior / final-year medical students; n = 99 | Prospective single-arm pre–post mixed-methods study | BRAVE generative AI-based breaking-bad-news simulator with reflective debrief and SPwICES-mapped feedback | Breaking-bad-news simulation following mandatory BBN course | Self-efficacy, willingness, intention to apply protocol, reflective debrief, user experience | None | Self-efficacy, willingness, and intention to apply the protocol improved significantly. Students perceived BRAVE as useful, realistic, safe, and feedback-rich. | Single-arm design; outcomes were self-reported. Written modality limited non-verbal cues, and occasional recognition errors were reported. |
| [29] | Haider et al. | 2025 | Canada | First- and second-year MD students; n = 15 | Mixed-methods feedback comparison study | ChatGPT-4 feedback on written narrative reflections | Professional identity reflection and formative feedback | Reflective learning, professional identity formation, feedback engagement; Likert ratings and open-ended responses | Longitudinal facilitator feedback | ChatGPT was rated higher for identifying strengths, areas for improvement, and actionable steps. Students still valued facilitator feedback for relational and personal depth. | Small convenience sample; ChatGPT feedback sometimes had an “AI tone” and paraphrased or misused quotations. Human relational context could not be reproduced. |
| [30] | Bauermann et al. | 2025 | Germany | Medical students; n = 82, including 66 second-semester and 16 fourth-semester students | Video-based simulation acceptance study | AI-based, avatar-mediated feedback and feedforward | Simulated doctor–patient consultation video | Communication feedback, feedforward, trustworthiness, learning acceptance, avatar perception | Feedback versus feedforward comparison within the AI-avatar condition | AI-based avatar-mediated feedforward was rated more trustworthy and more acceptable for learning than feedback. Avatar sympathy, human-likeness, trustworthiness, and attitudes toward AI influenced acceptance. | Participants rated feedback on a peer’s video rather than their own performance. The study assessed acceptance, not actual learning or behavioural change. |
| [31] | Ba et al. | 2024 | China | Paediatric trainees / medical interns; n = 77, ChatGPT-assisted n = 39 and traditional n = 38 | Controlled experimental study with blinded assessment | ChatGPT-4-assisted teaching | Two-week paediatric internship rotation | Patient communication, clinical judgement, professional conduct, clinical skills; Mini-CEX and theoretical examination | Traditional bedside teaching | The ChatGPT-assisted group showed better Mini-CEX performance, especially in patient communication and clinical judgement, while theoretical knowledge scores were similar. | Single-centre short intervention; long-term retention and clinical transfer were unclear. Outcomes may be influenced by implementation context and trainee acceptance. |
| [32] | Bentegeac et al. | 2025 | France | Undergraduate medical students from five medical schools; n = 91 | Multicentre prospective validation pilot study | ECOSBot, GPT-4o-powered standardised patient and examiner | Nephrology OSCE training across four clinical stations | OSCE practice, communication, professional attitude, feedback; authenticity, correctness, relevance, ICC, CUQ | Nephrology faculty gold-standard ratings | ECOSBot showed high fidelity as a standardised patient and strong agreement with human raters for global scores. Most students found the tool useful for OSCE preparation. | Communication and attitude ratings were less reliable. The study validated simulation and scoring performance rather than demonstrating educational outcome improvement. |
| [33] | Takahashi et al. | 2026 | Japan | Mixed clinical experience participants; n = 7, including medical students, residents, and attending physicians | Cross-sectional validation study | GPT-o1 Pro and GPT-5 Pro AI-based assessment of interview transcripts in an AI-simulated patient system | Clinical interview assessment using virtual patient transcripts | Assessment reproducibility, communication evaluation; Master Interview Rating Scale, reliability, agreement, assessment time | Human-based assessment by clinical instructors | AI-based assessment produced scores similar to human assessment, showed strong agreement, higher consistency, and reduced evaluation time by more than half. | Very small sample; single clinical case; transcript-based assessment excluded non-verbal behaviour and other contextual aspects of communication. |
| [34] | He et al. | 2026 | United States | Undergraduate nursing students; n = 23 | Qualitative study | Generative AI-enhanced virtual reality patient encounter simulation | Nursing patient encounter simulation | Communication, confidence, motivation, critical thinking, empathy-related learning; individual interviews | None | Students reported positive learning experiences and perceived the simulation as effective for practising patient encounters, communication, confidence, and critical thinking. | Technical issues were reported. Empathy-building was limited by robotic or synthetic voices and difficulty establishing emotional connection with virtual patients. |

**Note.** AI = artificial intelligence; AI-CST = artificial intelligence communication skills training; AI-VSP = artificial intelligence-based virtual standardised patient; BBN = breaking bad news; CUQ = Chatbot Usability Questionnaire; ICC = intraclass correlation coefficient; ITEM = Immersive Technology Evaluation Measure; LLM = large language model; Mini-CEX = Mini-Clinical Evaluation Exercise; OSCE = Objective Structured Clinical Examination; pre-CC OSCE = Pre-Clinical Clerkship Objective Structured Clinical Examination; SEGUE = Set the Stage, Elicit Information, Give Information, Understand the Patient’s Perspective, End the Encounter; SP = standardised patient; VIP = Virtual Integrated Patient.
